# Diagnosis of *O. volvulus* infection via skin exposure to diethylcarbamazine: clinical evaluation of a transdermal delivery technology-based patch

**DOI:** 10.1186/s13071-015-1122-9

**Published:** 2015-10-09

**Authors:** K. Awadzi, Nicholas O. Opoku, Simon K. Attah, Janis K. Lazdins-Helds, Annette C. Kuesel

**Affiliations:** Onchocerciasis Chemotherapy Research Centre, Hohoe, Ghana; Department of Microbiology, University of Ghana Medical School, Accra, Ghana; UNICEF/UNDP/World Bank/ WHO Special Programme for Research and Training in Tropical Diseases, World Health Organization, Geneva, Switzerland

**Keywords:** Africa, Onchocerciasis, Diethylcarbamazine patch, Diagnosis, Transdermal-delivery

## Abstract

**Background:**

Elimination of onchocerciasis in Africa is now regarded as an achievable goal in many areas. This makes monitoring changes in infection prevalence a key component of control programmes. Monitoring is currently based on determining the presence of *O. volvulus* microfilariae in skin snips, an invasive, labour-intensive method. The Onchocerciasis Control Programme (OCP) had established procedures to detect *O. volvulus* infections via the localized skin reaction induced by killing of microfilariae upon skin exposure to diethylcarbamazine via a patch (OCP-patch). Large scale OCP - patch use is difficult due to labour-intensive patch preparation. At the request of TDR, a manufacturer specialized in transdermal-delivery systems developed a ready-to-use diethylcarbamazine (DEC) containing patch (LTS-2 patch). To qualify this patch for large scale studies of its sensitivity and specificity, this study evaluated its ease of application, ability to detect infection and DEC exposure related adverse reactions compared to the OCP-patch in 30 infected individuals.

**Methods:**

Each participant with 0.2–36.8 *O. volvulus* microfilariae/mg skin received the OCP-patch and 4 days later the LTS-2 patch at the left and right iliac crest, respectively, for 24 h. Presence and characteristics of local skin reactions were assessed at patch removal and 6 h later. Skin reaction and Mazzotti reaction rates were compared with Fisher’s exact and a paired t-test, respectively.

**Results:**

The LTS-2 patch could be applied within 10 s. Mild itching occured at 63.3 % of OCP-patch (duration 8.9 ± 11.8 h) and 26.7 % of LTS-2 patch sites (duration 1.0 ± 2.5 h) and was the most frequent Mazzotti reaction. At patch removal after 24 h, a diagnostic local skin reaction was present under 90 % of OCP-patches and 83 % of LTS-2 patches; 6 h later, it was present at 93 % of OCP-patch and 100 % of LTS-2 patch sites.

**Conclusions:**

The data suggest that safety, tolerability and ability to detect infections of the LTS-2 patch are comparable to those of the OCP-patch. They qualify the LTS-2 patch for field studies to determine LTS-2 patch sensitivity, specificity and utility during large scale use and thus to inform use of the LTS-2 patch by onchocerciasis control programmes to determine prevalence of infection.

**Trial registration:**

Current controlled Trials ISRCTN76875372.

## Background

Onchocerciasis is caused by the filarial nematode *Onchocerca volvulus.* The parasite is transmitted between humans through blackfly vectors, in Africa mainly by *Simulium damnosum s.l.*. The African Programme for Onchocerciasis Control (APOC) estimated 89 million Africans at risk and 37 million infected in central and east Africa [1] based on rapid epidemiological mapping [2]. In 2010, the population in the APOC target area living in meso-endemic and hyperendemic areas was estimated to be 32.4 Million and 31 Million, respectively [3]. In the 13 endemic foci in 6 countries in Central and South America, the population at risk was estimated to be 0.56 million people living in an estimated 1939 communities of which 63 and 25 % were hypo- and meso-endemic, respectively. Since 2000, between 17–25 rounds of semi-annual treatment with ivermectin, complemented in 138 hyperendemic communities for several years by two additional treatments/year, have resulted or are likely to have resulted in interruption of transmission in 11 foci [4].

The feasibility of elimination of onchocerciasis through long term mass treatment with ivermectin in Africa has been demonstrated in Senegal and Mali [5, 6]. Significant reduction in prevalence of infection in children or adults after 5–13 years of annual community directed treatment with ivermectin has been shown in Cameroon, Uganda and Nigeria [7–9]. Consequently, the objectives of APOC have been expanded from control of onchocerciasis as a public health problem to elimination of onchocerciasis where feasible [10]. This requires large scale monitoring of the prevalence of current infection in areas undergoing mass treatment. Furthermore, there is a need to determine the prevalence of onchocerciasis in hypoendemic areas which are not yet included in onchocerciasis control activities to decide on the appropriate strategy to eliminate onchocerciasis. This requires diagnostic tools which are suitable for large scale use in communities.

Currently, the standard method for diagnosis of patent infection is “skin snipping”. This procedure demonstrates the presence of the microfilariae (mf) of *O. volvulus* in pieces of skin snipped (skin snips) from selected areas of the body. The results are quantifiable and are usually expressed as mf/mg skin in clinical studies where weighing of snips is feasible (see e.g. [11–13]) or mf/snip in field based studies (see e.g. [7, 14]). The main limitations of skin snipping are the need for trained personnel and special equipment (skin snip punches, microscopes, equipment for sterilization to avoid transmission of blood-borne pathogens and sometimes, field generators). Additional drawbacks are the inability to detect pre-patent and very light infections. Furthermore, communities and volunteers may accept the snipping only grudgingly or not at all. Thus, skin snipping is not suitable for large scale monitoring.

The presence of mf in the skin can be inferred by demonstrating the well-known events (Mazzotti reactions) that follow their destruction by diethylcarbamazine (DEC), such as itching, rash and lymphadenitis. Initial approaches used one oral 50 mg dose of DEC. This procedure, known as the Mazzotti test, is sensitive but yields a small number of both false positive and false negative reactions. A potential for false positive reactions is the presence of dermal mf other than *O. volvulus* that are sensitive to DEC (eg: *Mansonella streptocerca)*. The key aspects of the Mazzotti test have been summarised previously [15, 16].

A major limitation of the oral Mazzotti test is the potential for severe or even serious systemic adverse reactions to the killing of the microfilariae and ocular damage in heavily infected individuals [17–19]. The test is, therefore, recommended only for individuals in whom a diagnosis of onchocerciasis is strongly suspected but mf cannot be detected despite a detailed search in the skin and eyes [20, 21]. To avoid the systemic effects of oral administration, DEC has been applied topically to a small area of skin after dispersion in diverse excipients that facilitate drug penetration through the skin. The killing of mf is accompanied by a local skin reaction, a Mazzotti reaction, which has been validated as a surrogate marker of infection [22–24]. This diagnostic procedure is referred to as the “DEC patch test.” The Onchocerciasis Control Programme of West Africa (OCP) introduced a patch containing 20 % w/v DEC in a commercially available skin lotion (OCP-patch) as a safe, cheap, non-invasive and specific diagnostic test for monitoring *O. volvulus* infection [25–27]. A review of the experiences and considerations that led to the DEC patch test has been provided by Ozoh et al. [28].

Other diagnostic methods have been developed. Detection of *O. volvulus* DNA via polymerase chain reaction [29, 30] or *O. volvulus* antigens via immunoblotting [31] or a dip-stick assay [32] identifies current infection but requires tissue/blood samples, specialised laboratories, is expensive and not yet available for point-of-care use. Immunological assays based on antibody detection cannot differentiate previous from patent infections [33, 34] but will be valuable for surveillance for recrudescence of infection after the decision to stop ivermectin treatment in a transmission zone. A format to detect antibodies to the antigen Ov16 that does not require specialized laboratories and can be used as a ‘point-of-care’ diagnostic in the field was developed by PATH, funded by the Bill and Melinda Gates Foundation, and is now commercially available through Standard Diagnostics, Inc. (http://sites.path.org/dx/ntd/oncho/, http://www.path.org/news/press-room/703/ accessed 23 March 2015).

The main disadvantage of the OCP-patch is that it requires ad hoc preparation, i.e. dispersion of DEC in Nivea lotion that is then applied to a piece of filter paper and attached with a strip of adhesive plaster to the skin, a very labour intensive procedure for large scale use [26, 27]. Furthermore, neither concentration or homogeneity of the DEC containing lotion nor a standardized amount of the lotion on the filter paper can be assured. These disadvantages could be overcome by a DEC patch manufactured according to current ‘Good Manufacturing Practices’ [35] with a standardized DEC content and transdermal delivery capacity.

At the request of OCP and APOC, the UNICEF/UNDP/World Bank/WHO Special Programme for Research and Training in Tropical Diseases (TDR) approached research-based pharmaceutical groups specialized in drug transdermal delivery technologies for their willingness to develop a DEC patch for the onchocerciasis control programmes free of charge and in the context of lack of commercial potential of the patch. LTS Lohmann Therapie Systeme AG (LTS) was willing to develop a DEC patch in this context. The research and development programme conducted by LTS yielded a patch (LTS-2 patch) optimized for DEC content and skin permeation rates. In an *in vitro* system, approximately 50 μg/cm^2^ of DEC from the LTS-2 patch had permeated through human skin after 3 h and 500 μg/cm^2^ after 24 h (unpublished results).

The LTS development programme was complemented by a TDR sponsored clinical evaluation. The endpoints allowed the indirect assessement of whether the extent of DEC permeation into the skin from the LTS-2 patch was clinically significantly above or below that from the OCP patch: a significantly higher extent of DEC permeation would be indicated by significantly more frequent and/or more severe Mazzotti reactions and/or a local skin reaction extending further beyond the patch area than seen after the OCP-patch, while a significantly lower extent of DEC permeation would be indicated by a significant number of people with a local skin reaction after the OCP-patch, but no local reaction after the LTS-2 patch. Results from this clinical evaluation suggesting comparable DEC permeation would qualify the LTS-2 patch formulation for large scale evaluations of its sensitivity and specificity, otherwise, a new formulation of the patch would have to be developed with either increased or decreased DEC penetration into the skin. The clinical evaluation of the LTS-2 patch was conducted at the Onchocerciasis Chemotherapy Research Center (OCRC), Hohoe in Ghana.

This report describes the results of this evaluation in 30 participants known to be infected based on *O. volvulus* microfilariae detection in skin snips. The primary objective was to assess the safety and tolerability as well as the potential field utility (ease of application, presence and readout of diagnostic skin reaction) of the LTS-2 patch in comparison with the OCP-patch. Secondary objectives were (a) to compare the rate of positive skin reaction of the OCP-patch and the LTS-2 patch 24 and 30 h after application and (b) to determine the underlying pathology of the skin reactions from the histopathology of skin punch biopsies taken from selected participants.

## Methods

### Ethical approval and informed consent

The study protocol and informed consent documents were approved by the Ethics Committee of the Ghana Health Service and by the Secretariat Committee for Research Involving Human Subjects of the World Health Organization.

The information document that detailed all aspects of the study was read to and discussed with the community in the community in the local language (Ewe). Each participant signed or thumb printed an informed consent certificate approved by the Ethics Committees and received a copy of the informed consent document. An impartial witness and the investigator present during the meetings and discussions (KA or NOO) countersigned and dated the certificate.

The study took place at the OCRC, Hohoe, in Ghana in May and June 2006 and was conducted in accordance with good clinical practice, the Declaration of Helsinki (2000 revision) and all applicable local regulations.

### Study participants

Thirty men and women were recruited from onchocerciasis endemic communities in the basin of River Tordzi located in south-eastern Ghana (0° 30′ to 0° 45′ E, 6° 45′ to 7° 0′ N). During the study, ivermectin mass distribution had not yet been initiated because the area is overall hypoendemic with small meso- or hyperendemic foci [13]. Eligible participants were between 18 and 55 years with a body weight ≥ 40 kg and in good general health as determined by medical history, physical examination, electrocardiograms (ECGs) and clinical laboratory evaluations (see Table 1). The skin mf density determined as the arithmetic mean of the mf count/mg skin in one snip each from the right and left iliac crest was required to be low ( >0–20 mf/mg), since the primary concern was that the amount of DEC penetrating through the skin (≤6.2 mg, see below) would be insufficient to elicit detectable skin reactions. In contrast, the potential for severe reactions in multiple body systems as reported after systemic administration of DEC to highly infected people was not considered a concern. Studies of oral DEC administration to *O. volvulus* infected people have shown that the severity of Mazzotti reactions is related to the number of microfilariae killed, which depends on the intensity of infection pre-treatment and the dose of DEC [36–38]. The ≤ 6.2 mg DEC in the LTS-2 patches (see below) is substantially below the doses used for treatment of *O.volvulus* infected people (e.g. total doses of 100 to 6000 mg [37], single dose of 100 mg [17], daily doses of approximately 3 mg/kg/day for 7 days [39]). Subjects with microfilariae in the eyes, hyper-reactive onchodermatitis, skin lesions over the iliac crests, coincidental infection with *Mansonella streptocerca*, clinically significant ECG abnormalities, history of cardiac abnormalities or a history of alcohol or drug abuse were not eligible. The details of the procedures and laboratory tests conducted for assessing the eligibility of participants have been described previously [40] and are summarized in Table 1.

**Table 1:** Safety and efficacy evaluations

Study day	−5 to −1	1	2	3	4	5	6	7	8	9
Medical and medication history	X									
PE, height, 12-lead ECG	X									
Weight	X									X
Vital signs^(a)^	X	X	X	X	X	X	X	X	X	
Ocular examination^(b)^	X		X		X		X		X	
Haematology, serum biochemistry, urinalysis^(c)^	X				X				X	
Interim PE					X				X	
Adverse events		X	X	X	X	X	X	X	X	
Skin snip^(d)^	X				X				X	
Patch application		OCP				LTS-2				
Reaction readout^(e)^			X	X			X	X		
Skin punch biopsy			X				X			
Discharge										X
Day relative to OCP-patch application	−3	0	1	2	3	4	5	6	7	8
Day relative to LTS-2 patch application	−7	−4				0	1	2	3	4

*PE* physical examination

^(a)^Pulse rate, blood pressure, respiratory rate, and oral temperature measured after at least 5 min supine. 6 times/day during screening period, 2times/day on days 1–8

^(b)^Visual acuity, visual fields using a calibrated Goldmann perimeter, colour vision, external ocular structures, ocular mobility and pupillary reflex. Anterior segment examination with Haag-Streit 900 slit-lamp. Microfilariae in anterior chamber counted after head-down positioning for 5 min. Count of living and dead microfilariae in cornea and punctuate opacities. Intraocular pressure, dilated fundus examination with direct and indirect ophthalmoscopy. Fundus photography and fluorescein angiography was planned only on subjects with abnormalities of visual function (visual acuity or visual fields) and was not indicated in any subject

^(c)^Samples obtained in fasting state. Haematology: complete blood cell count (CBC), haematocrit, haemoglobin, WBC with manual differential, platelet count. Serum chemistry: sodium, potassium, chloride, carbon dioxide, glucose, total protein, albumin, blood urea, creatinine, alkaline phosphatase, lactic dehydrogenase (LDH), total bilirubin, gamma-glutamyl transpeptidase (GGT), aspartate aminotransferase (AST/SGOT), and alanine aminotransferase (ALT/SGPT). Urinalysis: 10-parameter dipstick (specific gravity, pH, protein, glucose, ketones, blood, bilirubin, urobilinogen, nitrite, leucocytes) and microscopic evaluation

^(d)^Two skin snips from right and left iliac crests using a 2 mm Holth or Walser-type corneoscleral punch. Each snip was weighed on an analytical balance and incubated overnight in isotonic saline in a well of a flat-bottomed microtitre plate. Emerged mf were counted under inverted microscope. The skin mf density at each site was calculated per mg skin. Screening skin snips were obtained on Day −3

^(e)^Local reaction reading on Day 2 and Day 6 occurred immediately after removal of the respective patch 24 h after its application and again 6 h later (i.e. 30 h after application of the respective patch and 6 h after its removal)

### Study design

This was an open label study of the OCP-patch and the LTS-2 patch. Each patch was applied once in each of the 30 participants. Subjects were admitted to the OCRC four to five days before the 1^st^ patch application and remained in the centre until 8 days afterwards. The OCP-patch was applied to the left iliac crest on day 1 and removed after 24 h. The LTS-2 patch was applied to the right iliac crest on day 5 and removed after 24 h. All examinations conducted and their timing are shown in Table 1.

### DEC patches and patch application

OCP-patch: The OCP-patch kit (provided by Dr. L. Toe, WHO Multi Disease Surveillance Center, Ouagadougou, Burkina Faso) consisted of 3 cm x 2 cm pieces of filter paper (Whatman 3MM; Whatman International, Maidstone, UK), 9 cm x 6 cm strips of perforated adhesive plaster, 25 g of diethylcarbamazine citrate salt (Sigma Chemical Corporation, St Louis, MO, USA) and 250 ml of Nivea skin milk (Beiersdorf, Norwalk, CT, USA). The patches were prepared according to the instructions provided with the kit [26, 27]. A 20 % DEC lotion (w/v) was prepared by thoroughly mixing 25 g of DEC salt and 125 ml of Nivea milk in a wide mouthed plastic container with a firm fitting cover. The container was labelled with the contents and date of preparation and kept in the refrigerator until used. As per the instructions in the kit, the solution is stable for 1 year in the refrigerator and for 6 months at room temperature in the shade. On the day of application, the patch was prepared and administered as follows: the lotion was left on a tray at 22–24 °C until it flowed freely out of the container and a small stainless steel gallipot was half filled with the solution. With a pair of dissecting forceps, a piece of filter paper was completely immersed into the DEC solution for around 10 s, removed and any excess DEC lotion eliminated by sliding both surfaces of the filter paper on the edge of the gallipot. The filter paper was placed in the middle of the adhesive surface of the perforated plaster that had been pre-labelled with the participant number on the opposite surface. The plaster was held by the edges and the longitudinal axis applied firmly to the left iliac crest previously swabbed with 70 % methylated spirit and allowed to dry. Based on the concentration of the DEC-nivea solution, the DEC molecular weight and the difference in weight of the dry and DEC solution soaked filter paper determined for 10 filter paper pieces, it was estimated that on average each filter paper contained approximately 40.8 mg of DEC salt.

LTS-2 patches, provided by Lohmann Therapie Systeme, are around 2.9 cm x 2.3 cm transparent films coated with 5.4 mg of DEC on one surface (acceptable range as per manufacturing specifications: 4.6 - 6.2 mg). The DEC coated adhesive surface is protected by two rectangular pieces of aluminized film of unequal size with bossed edges. Each patch was sealed in a white envelope labelled with the batch number. The envelopes were kept at a temperature of 22–24 °C. For LTS-2 patch application, the larger of the aluminized films over the DEC coated surface was pulled away, exposing part of the DEC coated surface. This was immediately applied to the skin previously swabbed with 70 % methylated spirit and allowed to dry. While holding the film securely to the skin with one hand, the smaller piece of aluminium foil was removed, exposing the rest of the DEC surface which was pressed onto the skin. The patch was then held firmly in position for a few seconds.

All patches were applied with 2 min between participants beginning at 0700 h. Subjects were advised not to bathe until after the removal of the DEC patch 24 h later.

At the time of this study, the stability data obtained by LTS supported stability for 12 months at 20–25 °C. Further stability studies conducted by LTS confirmed LTS-2 patch long term stability. In conjunction with the results of the study described here, presented to the Technical Consultative Committee of APOC in March 2007 [41], this allowed the epidemiological evaluations in Senegal and Mali in January-February 2008 [5] to use LTS-2 patches from the batch used in this study.

### Outcomes

Table 1 shows the timing and type of evaluations conducted.

Safety was assessed based on adverse events (AEs), i.e. unintended and unfavourable changes from pre-treatment [42] in clinical and ocular symptoms, vital signs and laboratory indices (haematological, biochemical and urine). Symptoms were documented whenever they occurred and during formal enquiries conducted twice daily throughout the admission period. Vital signs were recorded at the same time, and whenever an adverse event was reported. The assessment of seriousness was conducted based on the criteria in ICH guidelines [42] and grading of the severity of AEs and causality attribution employed modifications of previously published criteria [43, 44]. AEs that occurred on days 1 to 4 after OCP-patch application and on days 5 to 8 after the LTS-2 patch were regarded as potentially attributable to the patch applied. The decision on whether an AE was considered as related to the patch took into account the timing of AEs relative to patch application and the participant’s state of health as well as the known effects of exposure to DEC [17–21].

Presence of diagnostic local skin reactions: According to the OCP-patch manual [26, 27], the local reactions are read 24 h after patch application. In the study of the LTS patch prototype (unpublished data), several subjects complained of increased local itching approximately 6 h after removal of the OCP-patch. Therefore, the presence, type and intensity of the reaction at the site of the OCP-patch and of the LTS-2 patch were read 24 h and 30 h after patch application. Scoring of the intensity of the OCP-patch reactions was as described previously [25–27] (Table 2). Because the reactions under the LTS-2 patch site were different from those under the OCP-patch, a scoring system was developed for the LTS-2 patch (Table 2). KA and NOO scored the reactions jointly without disagreements recorded as having to be resolved.

**Table 2:** Scoring of OCP and LTS-2 DEC patch skin reactions

OCP DEC patch^a^		LTS-2 DEC patch	
Score	Criteria	Score	Criteria
0	No rash	0	No swelling; pin point papules only
1	1–3 discrete, well formed papules	1	<50 % of the patch application area is covered by swelling
2	4–8 discrete, well formed papules	2	≥50 % of patch application area is covered by swelling but an area without swelling is present
3	>8 discrete, well formed papules papules separated by normal skin	3	The outline of the patch is completely covered by a well defined swelling (the outline lesion)
4	Coarse or confluent papules or extensive oedema due to conglomeration of papules (peau d’orange)		

^a^OCP training manual [26, 27]

Potential DEC penetration into the tissue surrounding the patch area was assessed in two ways: the extent to which diagnostic rashes extended beyond the area under the patch and through skin mf densities in 1 skin snip obtained 1 cm from the patch application area on each of the two iliac crests on day 4 and day 8.

Presence of indicators of microfilarial death (microfilarial fragments, intraepithelial abscesses and eosinophils) under the patch application area: Skin punch biopsies of 4 mm diameter were taken 30 h after patch application from the area under the OCP-patch in two participants and from the area under the LTS-2 patch in eight participants. All skin punch biopsies were performed under aseptic conditions in the OCRC minor surgery theatre. The patch reaction site was cleaned with an antiseptic and methylated spirit, anaesthetized with 2 % xylocaine and a core of skin removed using a 4 mm skin biopsy punch (Stiefel Laboratorium GmbH, Mühlheimer Straße 231, 63075 Offenbach am Main, Germany). The biopsy site was secured with a single silk thread, covered with a sterile dressing and dressed until healed. The biopsy tissue was preserved in 10 times its volume of 4 % (w/v) buffered formaldehyde. All biopsies were provided at the same time to the parasitologist (trained in histopathology) in containers labelled only with the participant number. The parasitologist was thus blinded to the patch type. The biopsies were processed as for routine histopathology [45], stained with haematoxylin and eosin and six 4 μm sections examined.

### Statistical methods

The sample size of 30 participants provided a probability of >0.95 to detect at least 1 adverse event with a true frequency of ≥10 %.

Number analysed: All participants who qualified for the study received both patches and were included in the data analysis.

Safety analysis: The frequency and duration of adverse events occurring after the OCP-patch and after the LTS-2 patch and considered as related to patch administration by the investigator were compared using a paired t-test.

Local skin reaction analysis: The positive readout rates at 24 and 30 h for each patch were compared using Fisher’s Exact Test. The association between the positive readouts of the two patches was compared using McNemar’s test.

Skin mf densities at the two iliac crests pre-patch administration were compared with a paired t-test. Skin mf densities between skin snip sites and time points and between time points for each skin snip site were compared with a one way ANOVA considering the different skin mf counts from each participant as repeated measures (Tukey’s multiple comparison test).

Analyses were performed with PSPPIRE 7.10 (www.gnu.org) or GraphPad Prism version 6.02 for Windows (GraphPad Software, La Jolla California USA, www.graphpad.com).

## Results

### Participant flow and baseline characteristics

Thirty-seven volunteers gave informed consent and were screened. Fourteen women and 16 men were enrolled and completed the study as planned. Reasons for screen failure were: undetectable levels of skin mf (2), skin mf count > 20 mf/mg (4), microfilariae in the eye (1).

Table 3 shows the demographic and baseline characteristics of the 30 participants. The frequency of ocular symptoms present at baseline diminished over time without any intervention. Only 3/11 participants with ocular symptoms during screening had an ocular symptom (itching, discomfort (participants report they feel they have sand in the eyes without any evidence to that effect on ocular examination) or lacrimation) at the end of the study. There was no evidence of intraocular inflammation at any time during the study.

**Table 3:** Demographic and baseline characteristics of the 30 participants

Demographics	*n* (%)	Ocular symptoms	*n* (%)
Women	16 (53.3 %)	Itching	4 (13.3)
Mean^a^ (range) age women	44 (30–55)	Discomfort^c^	1 (3.3)
Mean^a^ (range) age men	34 (22–55)	Lacrimation	2 (6.7)
		Itching + lacrimation	1 (3.3)
		Itching + discomfort^c^	1 (3.3)
Microfilaremia	Across both crests	Left iliac crest	Right iliac crest
		OCP-patch site	LTS-2 patch site
Skin snip positive	30	30	27
Mean^a^ ± SD mf/mg skin	7.2 ± 7.8	7.7 ± 8.1	6.6 ± 9.5
Median	4.58	4.95	3.0
Range mf/mg skin^b^	0.2–36.8	0.3–31.8	0–47.7

^a^Arithmetic mean, ^b^Two subjects with a mean skin microfilariae density across both crests of > 20 mf/mg (21.5 mf/mg, 36.8 mf/mg) were included by mistake. ^c^Participants report a feeling as if they had sand in the eye without any evidence of particulate matter on ocular examination

During screening, all participants were skin snip positive at the left iliac crest, while three were negative at the right iliac crest. On day 4, two of these three participants were skin snip positive at both crests, the other was skin snip negative at both crests. There was no significant difference between the skin mf levels at the right and left iliac crest.

### Ease of application of LTS-2 patch

The LTS-2 patch was easily removed from its envelope with the gloved hand, and could be applied to the skin by an experienced operator within 10 s. In 5 out of 30 participants the LTS-2 patch detached before 24 h and was secured with strips of plaster.

### Adverse events considered not related to patch application

Events considered unreleated to patch application or study conduct by the investigators (KA and NOO) were recorded in 21 (70.0 %) of the participants, including in 10 only after OCP-patch application, in 4 only after LTS-2 patch application and in 7 after both patch applications. Events included ocular itching, discomfort or lacrimation (symptoms frequently observed in subjects infected with *O. volvulus*, see results of baseline investigations, Table 3), different types of pain (body, waist, epigastric, abdominal, chest, ear, head), pyuria, hematuria, bodily weakness, cough, and chills. These events were mild and none required intervention.

### Mazzotti reactions

The Mazzotti reactions (other than the diagnostic local skin reaction) are summarized in Table 4. No ocular inflammation or change in visual function occurred after either patch. Changes in laboratory values were not considered clinically significant by the investigators (KA and NOO). The most common Mazzotti reaction was itching. All itching was mild and resolved without treatment.

**Table 4:** Mazzotti reactions after OCP-patch and LTS-2 patch application

Mazzotti reactions	After OCP-patch	After LTS-2 patch
Itching at patch application site: *n* (%)^*^	19 (63.3 %) at OCP-patch site	6 (20.0 %) at LTS-2 patch site
		3 (10.0 %) at OCP-patch site^b^
Start (hrs after patch application)^a^ Mean ± SD	12.6 ± 13.4	6.9 ± 6.9 at LTS-2 patch site
Start (hrs after patch application)^a^ Range (Median)	0.8–52.0 (8.2)	1.5–19.4 (4.1) at LTS-2 patch site
		0.1, 0.5, 4.1 + 36.1 – at OCP-patch
Duration (hrs): Mean ± SD^a, **^	8.9 ± 11.8	1.0 ± 2.5 at LTS2 patch
Duration (hrs): Range (Median)	0–56 (7.63)	0–8.9 (0) – at LTS–2 patch
Duration (hrs)		1.1, 4, 6 + 12 – at OCP-patch
Itching involving the whole body: *n* (%)	5 (16.7)	2 (6.7 %)
Start (hrs after patch application)	4.1, 28.1, 51.9, 36.4, 4.3	5.7, 13.2
Duration (hrs)	7.9, 8.0, 8.2, 23.8, 55.9	6.3, 15.0
Itching at back	1 (3.3 %)	0
Other clinical Mazzotti reactions		
Burning sensation at patch site^c^	2 (6.7 %)	0
Pain at patch site	1 (3.3 %)	0
Haematological/biochemical reactions		
Leucocytosis with increase in peripheral granulocytes	4 (13.3)	6 (20.0 %)
Increase in AST	3 (10 %)^d^	0
Increase in ALT	3 (10 %)^d^	4 (13.3 %) + 2 (6.7 %^d^)
Increase in ALP	0	2 (6.7 %)

^*^ *p* = 0.0015, ^**^ *p* = 0.0014, ^a^descriptive statistics calculated across subjects with itching, ^b^includes 1 subject who had no itching at the OCP-patch site after the OCP-patch application, ^c^described by the participants as ‘pepper on the skin’, duration 3.3 and 6.4 h, resp. ^d^Elevations persisted into period after LTS-2 application and in 2 participants ALT elevation increased from grade 1 after OCP-patch application to grade 2 after LTS-2 patch application

**Fig. 1:**
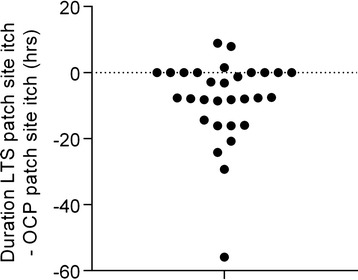
Difference in duration of patch site itching after LTS-2 and after OCP-patch for each participant. Each circle represents the difference in the duration of itching after the LTS-2 patch and the OCP-patch site for one study participant

Figure 1 shows the difference between the duration of the respective patch site itching after the LTS-2 patch and the OCP-patch for each subject. The frequency and the duration of patch site itching after the OCP-patch were significantly higher than after the LTS-2 patch (*p* = 0.0015 and *p* = 0.0014, respectively).

### Changes in ocular microfilariae over the course of the study

None of the participants had mf in the anterior chambers of the eyes at screening. One to three mf appeared in the anterior chamber of the eye of one participant on days 2, 4, 6 and 8; a single microfilaria appeared in the anterior chamber of the eye on day 2 in two participants and on day 6 and 8 in one participant each. This suggests that it is best to assume that microfilariae are present in the eye even if they are not visualized at the initial examination and skin mf densities are low.

### Characteristics of the local skin reaction under the patch sites

**Fig. 2:**
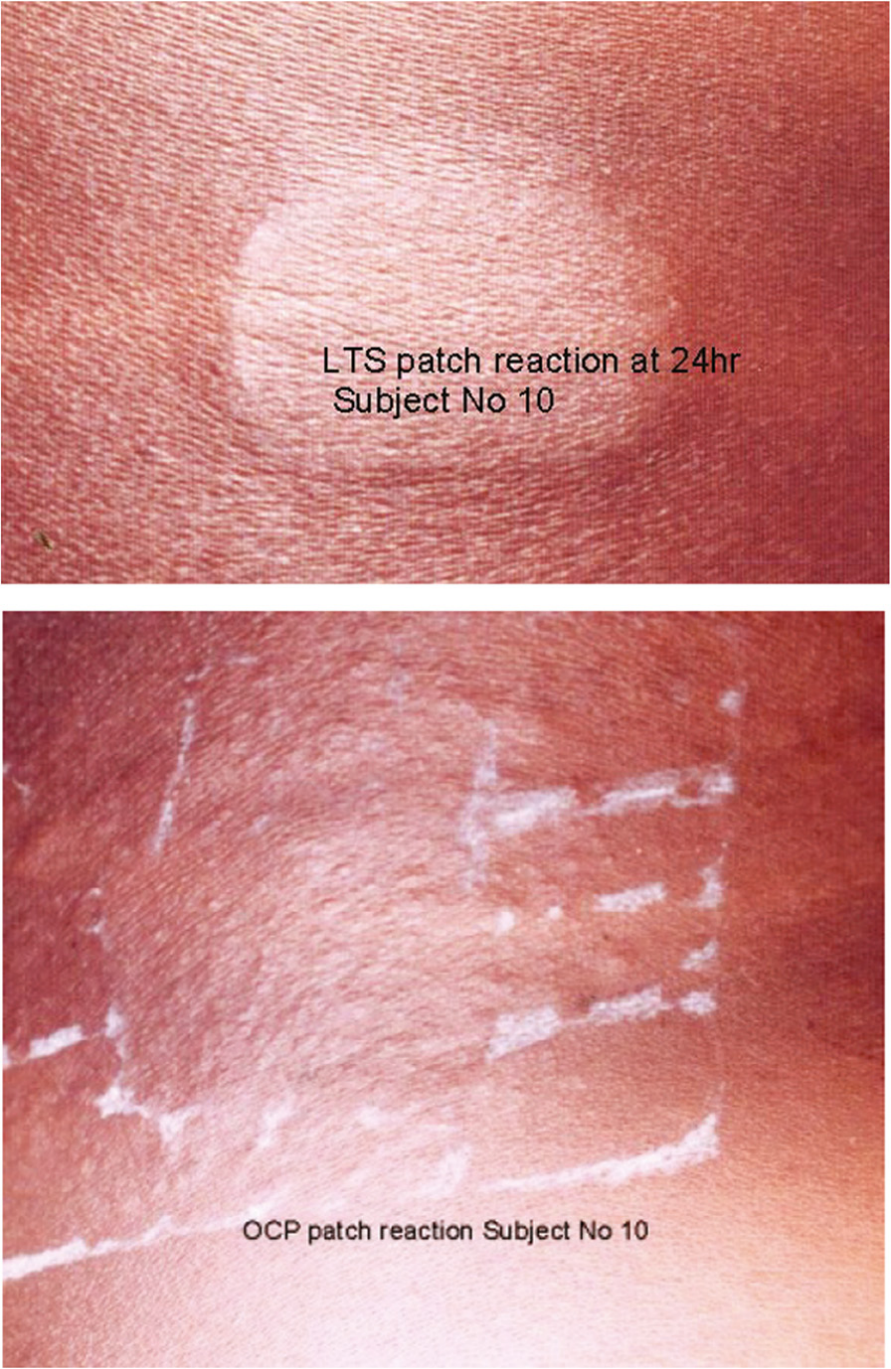
Local reaction under the LTS 2 patch application area (outline lesion, top) and under and beyond the OCP-patch (bottom) in the same subject

The OCP-patch reaction: In 19/30 (63.3 %) participants, the local reaction under the OCP-patch consisted mainly of well formed pinhead papules (Fig. 2 bottom) and could be scored based on the criteria reported previously [25–27]. These criteria are based on the counting of papules (scores 1–3) and the formation of a *peau d’orange* appearance caused by the coalescence of papules (score 4, Table 2). In 11/30 (36.7 %) participants, the reaction under the OCP-patch did not match the previously reported characteristics [25–27] and papules smaller than the pinhead papules (named ‘pinpoint’ papules) seen under the majority of OCP patches were observed. The characteristics of the reactions are summarized in Table 5. Three days after patch application, the skin reaction was still easily visible in 19 participants (63.3 %) and persisted in one participant 1 week after application.

**Table 5:** Number (%) of participants by characteristics of local reaction under the OCP and LTS-2 patches

Characteristics	OCP-patch (*N* = 30)		LTS-2 patch (*N* = 30)	
Definition of reaction	At 24 h	At 30 h	At 24 h	At 30 h
None	3 (10.0)	2 (6.7)	5 (16.7)	0
Oedema under the patch (any score)	NA	NA	25 (83.3)	30 (100)
100 % of area oedematous (score 3)	NA	NA	17 (56.7)	27 (90.0)
> 50 % of area oedematous (score 2)	NA	NA	4 (13.3)	3 (10.0)
< 50 % of area oedematous (score 1)	NA	NA	4 (13.3)	0
0 % of area oedematous (score 0)	NA	NA	5 (15.7)	0
All reactions	27 (90.0)	28 (93.3)	25 (83.3)	30 (100)
Very easily visible reaction	18 (60.0)	24 (80.0)	22 (73.	19 (63.3)
Easily visible reaction	2 (6.7)	3 (10.0)	0	1 (3.3)
Attentive visual inspection required	7 (23.3)	1 (3.3)	3 (10.0)	10 (33.3)
Character of papules				
None	3 (10.0)	2 (6.7)	5 (16.7)	0
Vague	4 (13.3)	1 (3.3)	3 (10.0)	0
Pinheads	16 (53.3)	16 (53.3)	0	0
Pinpoints	7 (23.3)	11 (36.7)	19 (63.3)	29 (96.7)
Urticariform	0	0	3 (10.0)	1 (3.3)
Extension beyond patch area	2 (6.7)	8 (26.7)	0	0

The LTS-2 patch reaction: The skin reaction which had developed under the LTS-2 patch sites did not consist of the pinhead papules typical for the OCP-patch reaction. Therefore, a detailed characterization of the reaction under the patch sites was performed ( Table 5).

The typical reaction, present in the majority of participants, presented as a well defined rectangular area that precisely matched the dimensions of the DEC impregnated area of the patch. This area was oedematous and studded with fine (pinpoint) papules (Fig. 2 top). This complex was termed the “outline lesion”. It resembled a “subdued” *peau d’orange*. In a minority of participants, not the whole area was oedematous (Table 5). The percentage of the area that is oedematous is the basis for the proposed scoring system (Table 2).

The intensity of the oedema also varied, being very intense or intense, and hence visible at ‘one glance’ (‘very easily visible reaction’ or ‘easily visible reaction’ in Table 5), or being less intense and requiring attentive visual inspection of the patch area for diagnosis. The intensity of the oedema is not part of the proposed scoring system: it adds an element with potentially high inter-observer variability and even with 100 % agreement between the two investigators (KA, NOO) in this study, did not improve the correlation between reaction score and skin mf levels (see below).

The oedema did not extend beyond the DEC impregnated area of the patch in any participant at either the 24 h or the 30 h reading.

The papules did not extend beyond the area covered by the patch either and did not conglomerate in any participant to form the “blood and thunder” oedema characteristic of the score 4 reaction after the OCP-patch. ‘Pinhead’ papules were not seen. Uncommonly, the papules were vague or shadowy and sometimes urticariform.

The outline lesion was present in 25 of 30 participants (83.3 %) at removal of the patch 24 h after administration. By 30 h, all participants were positive (Table 5). In four participants the reaction was less developed at 30 h than at 24 h, while in six others the reaction became better visible.

Three days after LTS-2 patch application, a skin reaction was visible in 5 (16.7 %) participants.

The proportion of participants with local reactions persisting to ≥ 3 days after patch application was significantly higher after the OCP-patch than the LTS-2 patch (*p* < 0.001).

### Skin microfilariae levels around 1 cm away from the patch application area

Skin mf levels around 1 cm from the patch application area were measured on Day 0 (i.e. before OCP-patch application, on Day 4 (i.e. 3 days after the OCP patch application and before LTS-2 patch application) and on Day 8 (i.e. 3 days after application of the LTS-2 patch). This was done with two objectives: a) to assess whether DEC penetrates beyond the patch application area in amounts significant enough to induce a reduction in skin mf levels (comparison of Day 0 and Day 4 measurement on the OCP-patch site for DEC penetration outside the OCP-patch area, comparison of Day 4 and Day 8 measurement for DEC penetration outside the LTS-2 patch area, and b) to estimate the number of skin mf levels for correlation with the patch scores (Day 0 and Day 4 skin mf levels for the OCP-patch and the LTS-2 patch, respectively).

**Fig. 3:**
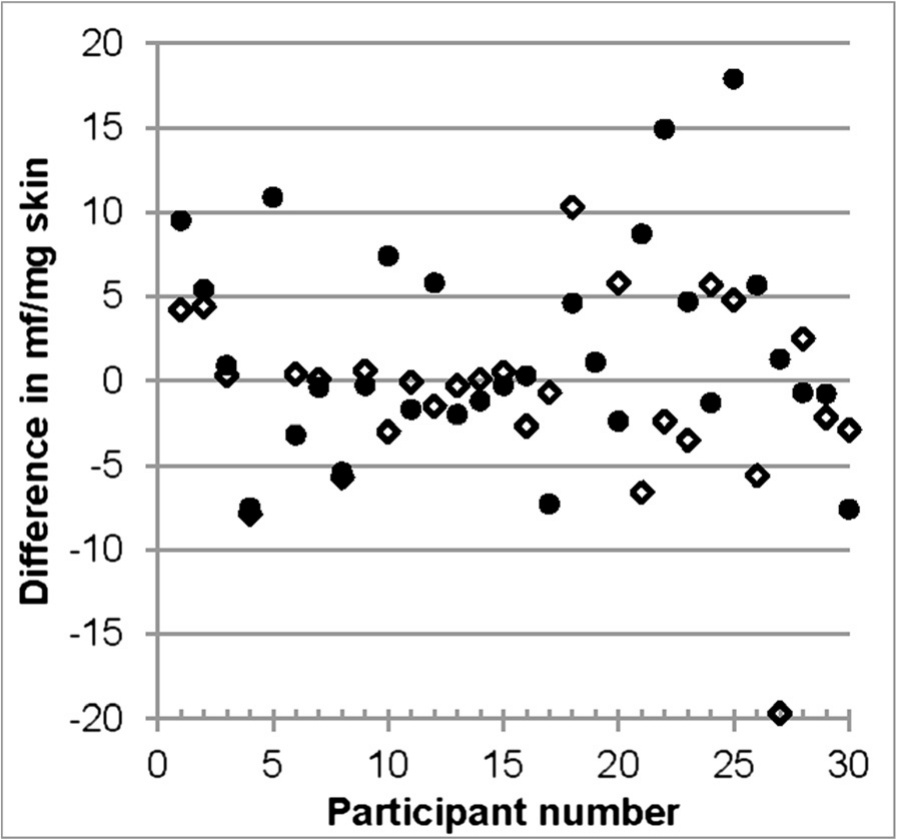
Differences in skin microfilariae levels from snips taken approximately 1 cm away from the patch application area taken 3 days after and one day before application of the respective patch. Solid circles: OCP patch area, open diamonds: LTS-2 patch area

For both patches, the mf levels in the snips taken after patch application were not consistently lower or higher than in the snip taken before patch application (Fig. 3). The descriptive statistics of the intra-individual differences between mf levels in snips taken before and after patch application calculated across all participants are shown in the lower panel of Table 6. These data suggest that the differences reflect local and/or temporal variation of mf density rather than changes induced by DEC present beyond the area exposed to the DEC in the patch. Consequently, the overall mean across all 6 snips taken from each participant is a better measure of the skin mf densities under the patch areas than are the mf/mg counts of the individual skin snips.

**Table 6:** Microfilariae counts in snips taken around 1 cm from patch application areas before and after the application of the OCP and LTS-2 DEC patches

	Left iliac crest (OCP-patch site)			Right iliac crest (LTS-2 patch site)		
	Microfilariae/mg skin			Microfilariae/mg skin		
Study day	Screening	Day 4	Day 8	Screening	Day 4	Day 8
Days relative to patch application	−3	3	7	- 7	−1	3
Mean ± SD^a^	7.73 ± 8.09	9.63 ± 9.92	6.63 ± 8.80^1^	6.61 ± 9.49	8.06 ± 9.55	7.44 ± 12.08
Range^a^	0.3 – 31.8	0.0 – 36.9	0.0 – 32.6	0.0 – 47.7	0.0 – 36.9	0.5 – 62.0
Lowest count eliciting local reaction at 24 hrs^a^	0.3	0	0.6	0	0	0.5
Highest count not eliciting local reaction at 30 h^a^	5.3	12.7	5.6	NA	NA	NA
Intra-individual difference in skin mf/mg	Day 4 – Screening	Day 8 - Screening	Day 8 – Day 4	Day 4 – Screening	Day 8 - Screening	Day 8 – Day 4
Mean difference^a^	1.90	−1.10	−3.00	1.45	0.83	−0.62
SD of difference	6.27	5.67	6.12	8.46	10.26	8.58
Median of difference	0.00	−0.35	−0.30	0.50	0.55	−0.20
Range of difference	−7.6–17.9	−11.8–10.5	−17.4–5.2	−28.3–18.8	−18.0–47.3	−22.1–28.5

^a^Across snips from the respective location and time from all 30 participants. Screening = Taken on admission and equivalent to day 0 for OCP-patch. Day 4 = 3 days after application of OCP-patch and day 0 for LTS-2 patch. Day 8 = 3 days after application of LTS-2 patch. *SD* standard deviation

^1^ *p* = 0.0228 for comparison of Day 4 and Day 8 skin mf level at the left iliac crest, all other comparisons *p* > 0.2

### Percentage of participants with local skin reactions and correlation between local reaction scores and skin microfilariae density

The percentage of participants with reactions at the 24 and 30 h reading was 90 % and 93 %, respectively for the OCP-patch and 83 % and 100 %, respectively, for the LTS-2 patch (Table 5). The percentage of positive reactions to the two patches was not significantly different at either time point (*p* = 0.71 for 24 h reading, *p* = 0.49 for 30 h reading).

**Fig. 4:**
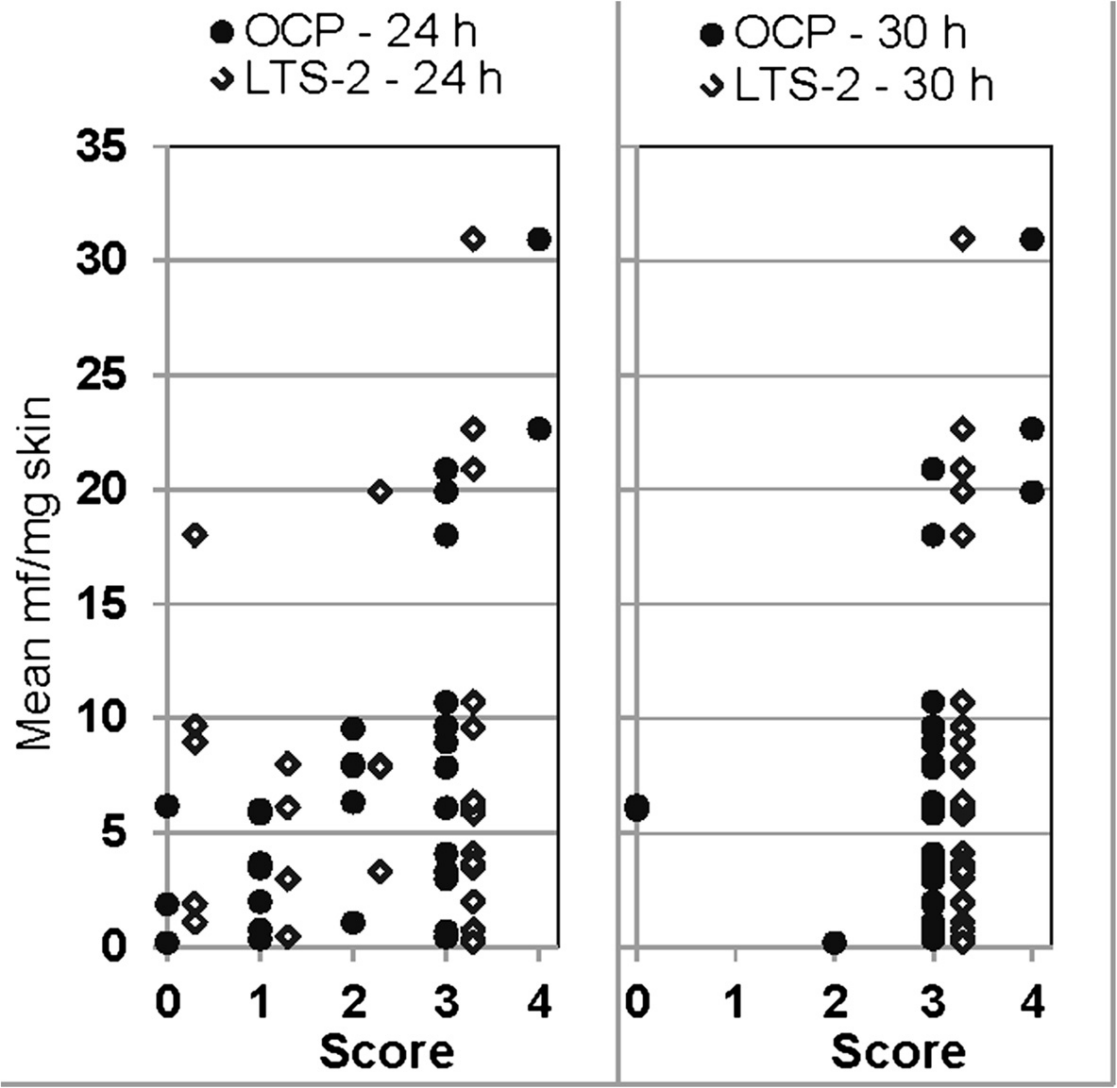
OCP-patch and LTS-2 patch scores read at 24 h and 30 h vs. mean skin mf count across all 6 snips taken

There was no correlation between the OCP-patch or the LTS-2 patch score at either reading time and the skin mf levels, whether the overall mean skin mf density was considered (Fig. 4, Table 7) or the skin mf count in the snip taken before patch application at the respective crest (Table 7).

**Table 7:** Proportion of subjects with local reaction by skin mf level measured 1 cm away from patch application area before application and by mean skin mf level across all skin snips taken

OCP-patch						LTS-2 patch					
Skin 1 cm from patch area pre application		% of subjects by score at 24 h		% of subjects by score at 30 h		Skin 1 cm from patch area pre application		% of subjects by score at 24 h		% of subjects by score at 30 h	
mf/mg	N	1	≥2	1	≥2	mf/mg	N	1	≥2	1	≥2
<1.0	5	20.0	40	0	100	<1.0	7	28.6	57.1	0	100
1–5	10	40.0	60	0	90.0	1–5	9	0	77.8	0	100
>5	15	13.3	80	0	93.3	>5	14	14.3	71.4	0	100
All snips						All snips					
mf/mg	N	1	≥2	1	≥2	mf/mg	N	1	≥2	1	≥2
<1.0	5	40.0	40.0	0	100	<1.0	5	20	80	0	100
1–5	8	37.5	50.0	0	100	1–5	8	12.5	71.4	0	100
>5	17	11.8	82.4	0	88.3	>5	17	11.8	70.6	0	100
Any	30	23.3	66.7	0	93.3	Any	30	13.3	70.0	0	100
		≥1		≥1				≥1		≥1	
Any	30	90		93.3		Any	30	83.3		100	

### Histopathology of skin punch biopsies from the reaction sites

A local skin reaction was deemed to be definitely due to the local death of mf if microfilarial fragments, intraepithelial abscesses and eosinophils were found in the biopsies. This was the case in the two biopsies taken after the OCP-patch (arithmetic mean skin mf density across all skin snips in these two participants were 22.7 and 30.9 mf/mg skin) and in 4 of the 8 biopsies taken after the LTS-2 patch application (arithmetic mean skin mf densities across all 6 skin snips from each of these four participants 4.1, 13.07, 17.4, 19.9 mf/mg skin). The presence of mf fragments without intraepithelial abscesses was regarded as probably due to local death of mf and seen in one of the biopsies taken from the LTS-2 patch site (arithmetic mean skin mf density across all skin snips 12.3 mf/mg). Presence of only eosinophils in the biopsy seen in 3/8 biopsies of the LTS-2 patch site (arithmetic mean skin mf levels across all 6 skin snips from each of these participants 0.9, 4.0, 7.7 mf/mg skin, reaction score 3 at both 24 and 30 h reading) was not regarded as evidence of local mf killing. Based on these criteria, evidence of local mf death was detected in 5 out of 8 biopsies taken after the LTS-2 patch.

## Discussion

The primary aim of this clinical evaluation was to determine the safety, tolerability as well as the potential field utility (ease of application, presence and readout of diagnostic skin reaction) for the diagnosis of current *O. volvulus* infection of the LTS-2 patch in participants known to be infected compared to the OCP-patch as indirect indicators of whether the transdermal delivery of DEC of the LTS-2 patch was clinically significantly different from that of the OCP-patch*.*

No adverse events other than Mazzotti reactions were assessed as DEC exposure related. The most frequent Mazzotti reaction was mild itching. Itching was significantly less frequent after the LTS-2 patch than after the OCP-patch. Mazzotti laboratory value changes were neither considered clinically significant nor significantly different between the two patches. The rate of the diagnostic local skin reaction was not significantly different between the OCP-patch and the LTS-2 patch. Together, these data suggest that DEC penetration into the skin with the LTS-2 patch is neither significantly higher nor diagnostically significantly lower than that with the OCP-patch, hence qualifying the LTS-2 patch for large scale field evaluations.

Oral DEC treatment can lead to a combination of very severe Mazzotti reactions in multiple systems whose cumulative effects are dangerous and alarming in people with high skin mf densities [37]. The severity of Mazzotti reactions correlates with the intensity of infection in patients treated with the same oral DEC dose, and correlates with the oral DEC dose when patients with similar mf levels are compared, i.e. the reaction severity correlates with the amount of mf killed [36, 37]. Thus, the safety profile of the LTS-2 patch in highly infected people potentially included in field evaluations and use depends on the total amount of mf killed after LTS-2 patch administration and the following needs to be considered: (1) the DEC dose, (2) the dose–response relationship for skin mf level reduction and (3) the number of mf that could be killed in highly infected people after LTS-2 patch application relative to the number of mf killed after oral DEC treatment of lightly infected patients in whom severe and dangerous Mazzotti reactions were not observed.

1. LTS-2 patch DEC dose relative to DEC doses used in oral treatment. Total oral DEC doses used were in the range of 100 mg to 6000 mg [37], single doses e.g. 100 mg [17], and daily doses e.g. 3 mg/kg/day for 7 days [39]. The maximum amount of DEC in a LTS-2 patch constitutes a total dose of only 6.2 mg DEC or, at a body weight of e.g. 30 kg, of only 0.2 mg DEC/kg for 1 day. If skin penetration in people does not exceed that in the *in-vitro* human skin assay used by LTS during pharmaceutical development (3 mg over 24 h, unpublished data), the total LTS-2 patch DEC dose available for systemic distribution is even lower. Any DEC becoming systemically available will be subject to the body’s elimination mechanisms. The DEC half-life after oral administration is around 9–10 h [46, 47]. Orally administered DEC is absorbed almost completely and quickly with peak concentrations 1–4 h after administration [46, 47]. In the *in vitro* human skin permeation assay, a total of around 0.3 mg, 1.1 mg and 3 mg had permeated across the total patch surface area by 3 h, 8 h and 24 h, respectively (unpublished results). These data suggest that, in contrast to absorption after oral administration, transdermal uptake is very slow which, in conjunction with ongoing DEC elimination, further reduces the maximum amount of DEC after the LTS-2 patch available for a systemic effect.
2. Dose–response curve for skin mf level reductions. The total oral DEC dose that results in a 90 % skin mf level reduction from baseline by 1 week after treatment was estimated at 500–600 mg. The dose–response curve is exponential with a total dose of 100 mg eliminating around 66 % of mf from the skin [37]. Even if the total 6.2 mg DEC in the LTS-2 patch became systemically available, it could consequently induce the death of only a small fraction of mf. The skin snips obtained approximately 1 cm from the patch areas did not suggest that the LTS-2 patch (or the OCP-patch) led to DEC concentrations outside the patch area sufficient to result in measurable skin mf level reductions (Table 6, Fig. 3).
3. Number of mf under the LTS-2 patch area in highly infected individuals relative to the number of mf killed after oral DEC treatment in lightly infected people in whom dangerous Mazzotti reactions have not been reported. Assuming a highly infected person with an average skin mf density of 2000/snip (i.e. approximately 2000 mf/4 mm^2^ skin surface area for snipping with a 2 mm Holth punch), 300,000 microfilariae are under the 6 cm^2^ DEC-impregnated LTS-2 patch area whose death could be induced. For comparison, assuming a very lightly infected person with an average skin mf density of only 1/snip (1/4 mm^2^ skin surface area), and a body surface area of 1.0 m^2^ (30 kg, 123 cm) or 1.7 m^2^ (60 kg, 171 cm), the number of skin mf that could be killed by oral DEC treatment is 250,000 and 425,000, respectively.

These considerations suggest that the number of mf killed after LTS-2 patch application in highly infected people is unlikely to exceed the number of mf killed after oral DEC treatment of lightly infected people in whom severe Mazzotti reactions in multiple systems have not been observed. Consequently, development of dangerous reactions when the LTS-2 patch is used in highly infected people is very unlikely, while more severe itching and rash than observed in this study is likely.

Application of the LTS-2 patch was easy and can be done within 10 s. Five out of 30 (16.7 %) LTS-2 patches detached before the 24 h reading. During use of the LTS-2 patch in the epidemiological evaluations in Mali and Senegal up to one third of the LTS-2 patches had partly or completely detached before the reading of the reaction at 24 h [5]. The detachment rate differed between countries (0/775 (0 %) in Senegal, 382/1508 (25.3 %) in Mali, Hans Remme, report to the Technical Consultative Committee of APOC). The LTS-2 patch uses a pressure-sensitive adhesive. It is not possible to assess now whether variability in the duration and/or firmness with which the patches were applied contributed to the variable detachment rates. Training for future use will have to stress the importance of the subject applying light pressure for 10 s after application of the LTS-2 patch and the difference between application of this and a conventional plaster. Reinforcement of patch adhesion with a strip of conventional plaster as recommended in the OCP-patch manual [26, 27] could also be considered for large scale LTS-2 patch use.

The skin reaction under the LTS-2 patch was very different from that under the OCP-patch. Based on the data from this study, the reading of the LTS-2 reaction is best done by examination for the presence of the pinpoint papule-studded oedematous area under the patch. The significance of the pinpoint papules is currently unknown and therefore the presence of only pinpoint papules without oedema is not regarded as indicative of the presence of microfilariae (score 0, Table 2). None of the 2116 skin snip negative participants examined in Mali and Senegal who were also examined via the LTS-2 patch had a skin reaction [5]. This suggests that the LTS-2 patch does not elicit a local reaction in the absence of skin microfilariae. Training (as conducted by OCRC staff before LTS-2 patch use in the epidemiological evaluations in Senegal and Mali) and attention to detail during the reading of the reaction will be important to ensure recognition of all LTS-2 reactions to avoid false negatives.

In 12/30 (40 %) of participants the LTS-2 score increased from 0, 1 or 2 at the 24 h to 3 at the 30 h reading. Ten of these 12 participants (83 %) had a mean skin mf level across all skin snips taken of < 10 mf/mg. Increase in scores from the 24 h to the 30 h examination were also seen with the OCP-patch, consistent with previous reports of higher sensitivity (% of positive reactions in infected participants) and intensity of reaction at 48 h than 24 h reading [25].

The underlying basis of the skin reactions was examined through skin punch biopsies from 2/30 OCP-patch and 8/30 LTS-2 patch sites. In 3/8 biopsies from LTS-2 patch sites no intra-epidermal abcesses and/or microfilariae fragments supporting a DEC effect on local microfilariae were detected. These three biopsies were from participants with low skin mf levels (mean of <8 mf/mg skin across all 6 skin snips taken). The commensurate low density of any microfilariae fragments that may have been present under the patch area may have been below the detection limit achievable with examination of six 4 μm slices from a 4 mm diameter punch biopsy. Further biopsy studies need to be undertaken to better define the pathogenesis of the local reaction and to assess the potential for false positives.

Large scale evaluation with 24 h and 30 h readings in a population with low levels of skin microfilariae (e.g. >0 to 10 mf/mg), ideally from an area co-endemic for other filarial species and incorporating other field-suitable and, if feasible, ‘research only’ tests for detection of current and past *O. volvulus* infection (e.g. OV 16 antibody detection, detection of *O. volvulus* DNA in skin snips via polymerase chain reaction), are recommended to define the sensitivity of the LTS-2 patch and to determine its specificity. The data will provide the basis for onchocerciasis control programme decisions on how to integrate the LTS-2 patch into monitoring of prevalence of *O. volvulus* infection, in particular residual prevalence in areas under long term CDTI where it is important to diagnose only active infection. Large scale evaluation of the LTS-2 patch in areas with different levels of prevalence of *O. volvulus* infection was also recommended by the APOC Technical Consultative Committee when it advised APOC to set up an agreement with LTS to provide the LTS-2 patches at low cost [48]. The LTS-2 patch is not available commercially but manufactured by LTS at the request of WHO and provided to WHO at cost. Countries can request patches from WHO.

## Conclusions

The results from this study suggest that the LTS-2 patch has a safety and tolerability profile similar to and possibly better than that of the OCP-patch and that it elicits local skin reactions in a comparable proportion of patently infected individuals. This suggests that the transdermal delivery of DEC by the LTS-2 patch is comparable to that of the OCP-patch which qualifies the LTS-2 patch for large scale evaluations to determine its sensitivity and specifity.
